# Berberine ameliorates chronic intermittent hypoxia‐induced cardiac remodelling by preserving mitochondrial function, role of SIRT6 signalling

**DOI:** 10.1111/jcmm.18407

**Published:** 2024-06-18

**Authors:** Zijun Zhou, Qiusheng Zhao, Yuting Huang, Shan Meng, Xin Chen, Guoxin Zhang, Yanbang Chi, Dengyue Xu, Zongtao Yin, Hui Jiang, Liming Yu, Huishan Wang

**Affiliations:** ^1^ State Key Laboratory of Frigid Zone Cardiovascular Disease, Department of Cardiovascular Surgery General Hospital of Northern Theater Command Shenyang Liaoning China; ^2^ Shenyang Joint Logistics Support Center Pharmaceutical Instruments Supervision and Inspection Station Shenyang China; ^3^ Jinzhou Medical University Jinzhou Liaoning China; ^4^ School of Biomedical Engineering, Faculty of Medicine Dalian University of Technology Dalian Liaoning China

**Keywords:** berberine, cardiac remodelling, chronic intermittent hypoxia, mitochondrial function, oxidative stress, SIRT6

## Abstract

Chronic intermittent hypoxia (CIH) is associated with an increased risk of cardiovascular diseases. Previously, we have shown that berberine (BBR) is a potential cardioprotective agent. However, its effect and mechanism on CIH‐induced cardiomyopathy remain uncovered. This study was designed to determine the effects of BBR against CIH‐induced cardiac damage and to explore the molecular mechanisms. Mice were exposed to 5 weeks of CIH with or without the treatment of BBR and adeno‐associated virus 9 (AAV9) carrying SIRT6 or SIRT6‐specific short hairpin RNA. The effect of BBR was evaluated by echocardiography, histological analysis and western blot analysis. CIH caused the inactivation of myocardial SIRT6 and AMPK‐FOXO3a signalling. BBR dose‐dependently ameliorated cardiac injury in CIH‐induced mice, as evidenced by increased cardiac function and decreased fibrosis. Notably, SIRT6 overexpression mimicked these beneficial effects, whereas infection with recombinant AAV9 carrying SIRT6‐specific short hairpin RNA abrogated them. Mechanistically, BBR reduced oxidative stress damage and preserved mitochondrial function via activating SIRT6‐AMPK‐FOXO3a signalling, enhancing mitochondrial biogenesis as well as PINK1‐Parkin‐mediated mitophagy. Taken together, these data demonstrate that SIRT6 activation protects against the pathogenesis of CIH‐induced cardiac dysfunction. BBR attenuates CIH‐induced myocardial injury by improving mitochondrial biogenesis and PINK1‐Parkin‐dependent mitophagy via the SIRT6‐AMPK‐FOXO3a signalling pathway.

## 1 INTRODUCTION

As a common disorder in the general population, obstructive sleep apnoea (OSA) is characterized by the collapse of the upper airway and chronic intermittent hypoxia (CIH) during sleep.^1^ OSA‐associated CIH is recognized as an independent risk factor for cardiovascular diseases including atherosclerosis, hypertension, coronary heart disease, myocardial infarction and arrhythmias.^2^, ^3^ Previous studies have revealed that CIH increases the risk of cardiovascular disease owing to the cardiac structural remodelling and cardiac dysfunction by triggering oxidative stress and myocardial fibrosis.^4^ Nevertheless, the current understanding of the molecular mechanism of CIH‐induced cardiac dysfunction remains limited.

The sirtuin (SIRT) family (SIRT1‐SIRT7) is a group of conserved nicotinamide adenine dinucleotide (NAD^+^)‐dependent class III histone deacetylases. It has been shown that sirtuin family members are widely distributed in the nucleus, cytoplasm and mitochondria, and play vital roles in maintaining intracellular homeostasis.^5^ Previous studies have demonstrated that the sirtuin family members are involved in the pathological condition of intermittent hypoxia.^6^, ^7^, ^8^ SIRT6, a member of the sirtuin family, is a nuclear protein primarily responsible for gene expression control and DNA repair. In the last decade, SIRT6 has been recognized to play multiple functions in stress resistance, apoptosis, aging, senescence, and inflammation.^9^ Notably, studies by our team and others have demonstrated that SIRT6 serves as a critical modulator in protecting cardiomyocytes against ischemia/reperfusion injury by augmenting antioxidant defence mechanisms and improving mitochondrial quality control.^10^, ^11^, ^12^ However, the relationship between CIH‐induced cardiomyopathy and SIRT6 remains uncovered.

Berberine (BBR) is a major form of isoquinoline alkaloid (Figure 2A) isolated from medicinal plants such as *Hydrastis canadensis* and *Rhizoma coptidis*. Due to its powerful antimicrobial, antioxidant and anti‐inflammatory activities, numerous studies have reported the pharmaceutical value of BBR in digestive system, immune system, nervous system and critically, cardiovascular diseases.^13^, ^14^ Our group and others have shown that BBR plays a therapeutic role in myocardial damage caused by multiple causes.^15^, ^16^, ^17^ In particular, studies have shown that SIRT1 and SIRT3 play a critical role in the cardioprotective effects of BBR.^18^, ^19^ However, the protective effect of BBR on CIH‐induced cardiac remodelling and myocardial oxidative stress remain unknown. In addition, it is not yet clear the potential role of SIRT6 in these effects.

Therefore, this study was designed to investigate the role of SIRT6 during the progression of CIH‐induced cardiac damage. In addition, we evaluated whether BBR conferred a protective effect against CIH‐induced cardiac remodelling and oxidative stress. Next, we explored the molecular mechanisms with a focus on SIRT6‐adenosine 5′‐monophosphate‐activated protein kinase (AMPK)‐ forkhead box O3alpha (FOXO3a) signalling and mitochondrial quality control processes.

## 2 MATERIALS AND METHODS

### 2.1 Gene ontology (GO) and kyoto encyclopedia of genes and genomes (KEGG) analysis

For functional enrichment analysis, an expression profiling dataset of the CIH and control groups (GSE138008) was screened from the Gene Expression Omnibus (GEO) database (https://www.ncbi.nlm.nih.gov/geo/).^20^ The series matrix files of GSE138008 were downloaded and processed with the ‘limma’ R packages by employing R platform (version 4.2.1). Differentially expressed genes (DEGs) were identified using the ‘limma’ package (absolute Log_2_FC value greater than 0.263, a *p*‐value <0.05).^21^ We performed GO and KEGG pathway enrichment analyses for these DEGs using the Metascape bioinformatics resources (http://metascape.org, version 3.5.20230501).

### 2.2 Animals and treatments

Male C57/BL6 mice (8 weeks of age) were obtained from Beijing HUAFUKANG Bioscience Co., LTD (Beijing, China). In the present study, the experiment was conducted in strict compliance with Institutional Animal Care and Use Protocols and approved by the Animal Care Committee of the General Hospital of Northern Theatre Command (No. 2021–14, 17 December 2021). Mice were randomized into five groups (15 animals per group) as following: Control group, CIH group, CIH + 50 mg/kg BBR (BBR L) group, CIH + 100 mg/kg BBR (BBR M) group, CIH + 200 mg/kg BBR (BBR H) group.

BBR (Figure 2A, purity ≥95%, B139120, Aladdin, Shanghai, China) was dissolved in the water and administered orally to the mice. The dose of BBR was chosen based on the preliminary experiment in our group and previous publications by our team and others.^17^, ^19^, ^22^ Hypoxic chambers (ProOx‐100; TOW‐INT TECH, Shanghai, China) were used to establish the CIH mouse models. The O_2_ concentration in these chambers was decreased from 21 to 5–8% by injection of N_2_ and then maintained for 15 s, followed by a rapid increase in O_2_ concentration to 21% for 60 s. The cycle was repeated for 12 h daily for 5 weeks. Mice in the control group were exposed to normoxic air at all times.^23^, ^24^ All the animals survived throughout the experimental period.

### 2.3 Adeno‐associated virus infection and experimental design

The recombinant adeno‐associated virus 9 (AAV9) carrying a cardiac troponin T (c‐TNT) promoter that expressed mouse SIRT6 or SIRT6‐specific short hairpin RNA (shRNA) were constructed by Hanbio Co., Ltd (Shanghai, China). The mice were randomly allocated to the following groups (15 animals per group): (1) control + AAV9‐NC group (CON‐NC); (2) CON+BBR (100 mg/kg) + AAV9‐NC group (CON‐BBR); (3) CIH + AAV9‐NC group (CIH‐NC); (4) CIH + BBR (100 mg/kg) + AAV9‐NC group (CIH‐BBR); (5) CIH + BBR (100 mg/kg) + AAV9‐shRNA SIRT6 group (CIH‐BBR‐shRNA SIRT6); (6) CIH + AAV9‐SIRT6 group (CIH‐SIRT6). All the animals survived throughout the experimental period. The virus titre of the AAV9 virus suspension was >10^12^ vg/mL, and mice were injected with 100 μL of the solution via the tail vein as described previously.^25^, ^26^

### 2.4 Evaluation of body index and hemodynamic parameter

The mice and their heart mass were weighted on an electronic scale at the end of the experimental period. Hemodynamic parameters and resting heart rate (HR) of each group were measured using a small animal blood pressure meter (BP‐2010A; Softron Beijing Biotechnology Co., Ltd, Beijing, China). The blood pressure meter utilized a pressure recording sensor and a tail cuff for the non‐invasive evaluation of systolic and diastolic blood pressure in mice as described previously.^27^

### 2.5 Echocardiography

Cardiac function was assessed using a high‐resolution doppler ultrasound imaging system for small animals (D700; Vinno Technology Co., Ltd, Suzhou, China) as described previously.^28^ Blind assessment was ensured during the measurements. Ventricular structure was recorded in the parasternal long‐axis view. Heart rate, left ventricular ejection fraction (LVEF), left ventricular fractional shortening (LVFS) and left ventricular end‐diastolic posterior wall thickness (LVPWd) were calculated from the recorded M‐mode images using a pre‐set computer program.

### 2.6 HE and Masson's trichrome staining

For histological analysis, the mouse hearts were harvested and briefly perfused with 10% potassium chloride.^29^ Then, the samples were fixed in 4% paraformaldehyde and embedded in paraffin. Paraffin‐embedded samples were cut into 5 μm sections for HE and Masson's trichrome staining. H&E staining (KGA224; KeyGEN Biotech, Jiangsu, China) was used to observe histomorphological changes. Masson's trichrome staining (abs9348; Absin, Shanghai, China) was used to assess cardiac fibrosis. Image J software (Image Solutions, Torrance, CA, USA) was used to quantify the data.

### 2.7 Immunohistochemistry

Myocardial SIRT6 levels were determined by immunohistochemical staining. Cardiac paraffin‐embedded tissues were sectioned at 5 μm thickness. Sections were subjected to heat‐mediated antigen retrieval, peroxidase blocking with 3% hydrogen peroxide and non‐specific binding site blocking. All slides were incubated with anti‐SIRT6 antibody (1:200 dilution; #12486, Cell Signalling Technology, MA, USA) at 4°C overnight. The sections were then treated with horseradish peroxidase‐conjugated antibodies and detected with 3,3′‐diaminobenzidine (DAB, KeyGEN BioTECH, Jiangsu, China). 5 random fields were captured by using a stereomicroscope (#UB2031; UOP Technology, Chongqing, China). SIRT6 levels were analysed and calculated using Image J software (Image Solutions, Torrance, CA, USA).

### 2.8 Transmission electron microscopy

The procedures were performed as previously described.^28^ Briefly, ventricular samples were initially fixed with 2.5% glutaraldehyde and then post‐fixed in 1% osmium tetroxide. The cardiac ultrastructure was captured and analysed in a blinded manner with a transmission electron microscope (OLYMPUE, Tokyo, Japan).

### 2.9 Immunofluorescence

Cross‐sectional area of the cardiomyocytes was determined by analysing immunofluorescence images of wheat germ agglutinin (WGA) staining. Briefly, frozen sections (4 μm) were incubated with 10 μg/mL WGA solution (W11261; Invitrogen, Carlsbad, CA, USA) at 37°C for 30 min, and the nuclei were stained with DAPI solution (Beyotime Biotechnology, Shanghai, China) for 15 min at room temperature. Fluorescence images were taken in 5 random fields from each section. α‐SMA and CD31 was evaluated by immunofluorescence staining. The 4‐μm‐thick paraffin ventricular sections were blocked in 0.01 M PBS containing 0.2% Triton X‐100 and 5% bovine serum albumin. Then, the sections were incubated with the following primary antibodies at 4°C overnight: CD31 (1:200 dilution, #77699S; Cell Signalling Technology, USA), α‐SMA (1:200 dilution, #48938S; Cell Signalling Technology, USA). The nuclei were stained with DAPI solution (Beyotime Biotechnology, Shanghai, China) for 10 min. Fluorescence images were captured by using a Nikon C2 Plus confocal microscope (Nikon, Tokyo, Japan).

ROS generation was determined using a dihydroethidium (DHE) assay kit (S0063, Beyotime Biotechnology, Shanghai, China) and a MitoSOX Red mitochondrial superoxide indicator (M36008; Invitrogen, UK). The nuclei were stained with DAPI solution (Beyotime Biotechnology, Shanghai, China). Fluorescence images were captured in 5 random fields using a Nikon C2 Plus confocal microscope (Nikon, Tokyo, Japan). All data were analysed and calculated using Image J software.

### 2.10 Assessment of glutathione/glutathione disulphide (GSH/GSSG) ratio

To assess cellular redox metabolism, the GSH/GSSG ratio was determined using commercially available kits (S0053; Beyotime Biotechnology, Shanghai, China) according to the manufacturer's instructions.

### 2.11 Western blot

The total protein content of ventricular samples was extracted using a lysis buffer containing protease inhibitor cocktail.^30^ The samples were lysed on ice for 30 min and centrifuged at 12,000 rpm for 30 min. Then, the protein concentration was determined by BCA method (P0012; Beyotime Biotechnology, China). Sodium dodecyl sulphate‐polyacrylamide (SDS‐PAGE) gel was used to isolate the protein, which was then transferred to a polyvinylidene fluoride (PVDF, 0.45 μm) membrane (IPVH00010; Millipore Corporation, Billerica, MA, USA). The membranes were blocked with 5% skim milk for 1.5 h and incubated overnight at 4°C using the primary antibodies, including SIRT6 (1:1000 dilution, #12486; Cell Signalling Technology, USA), AMPK (1:1000 dilution, #5831; Cell Signalling Technology, USA), p‐AMPK (1:1000 dilution, #2535; Cell Signalling Technology, USA), FOXO3a (1:1000 dilution, #9465; Cell Signalling Technology, USA), COL1A1 (1:1000 dilution, ab260043; Abcam, MA, USA), COL3A1 (1:500 dilution, sc‐271,249; Santa Cruz Biotechnology, USA), α‐SMA (1:1000 dilution, #19245; Cell Signalling Technology, USA), TGF‐β (1:500 dilution, sc‐130,348; Santa Cruz, USA), SOD2 (1:1000 dilution, #13141; Cell Signalling Technology, USA), Catalase (1:1000 dilution, #14097; Cell Signalling Technology, USA), Parkin(1:1000 dilution, #4211; Cell Signalling Technology, USA), PINK1 (1:500 dilution, sc‐517,353; Santa Cruz, USA), VEGF (1:500 dilution, sc‐7269; Santa Cruz Biotechnology, USA), PGC1α (1:500 dilution, sc‐518,025; Santa Cruz Biotechnology, USA), NRF‐1 (1:500 dilution, sc‐28,379; Santa Cruz Biotechnology, USA), TFAM (1:500 dilution, sc‐166,965; Santa Cruz Biotechnology, USA) and GAPDH (1:500 dilution, sc‐365,062; Santa Cruz Biotechnology, USA). Then, the membranes were incubated with the corresponding secondary antibodies for 1.5 h at room temperature. The protein bands were detected by Tanon image analyser (Tanon‐5200; bioTanon, Shanghai, China).

### 2.12 Data analysis and statistics

All data are presented as mean ± standard deviation (SD). Differences between the groups were compared using the Student's *t*‐test (for two groups) or one‐way analysis of variance (ANOVA) followed by the Turkey's comparison test was used to assess the differences among multiple groups. The statistical analyses were performed with the Prism software (GraphPad Prism 8.0; GraphPad Software Inc., CA, USA). The statistical significance was defined as *p* < 0.05.

## 3 RESULTS

### 3.1 Mitochondrial dysfunction and impaired SIRT6 signalling may play critical roles in the pathogenesis of CIH‐induced cardiac injury

**FIGURE 1:**
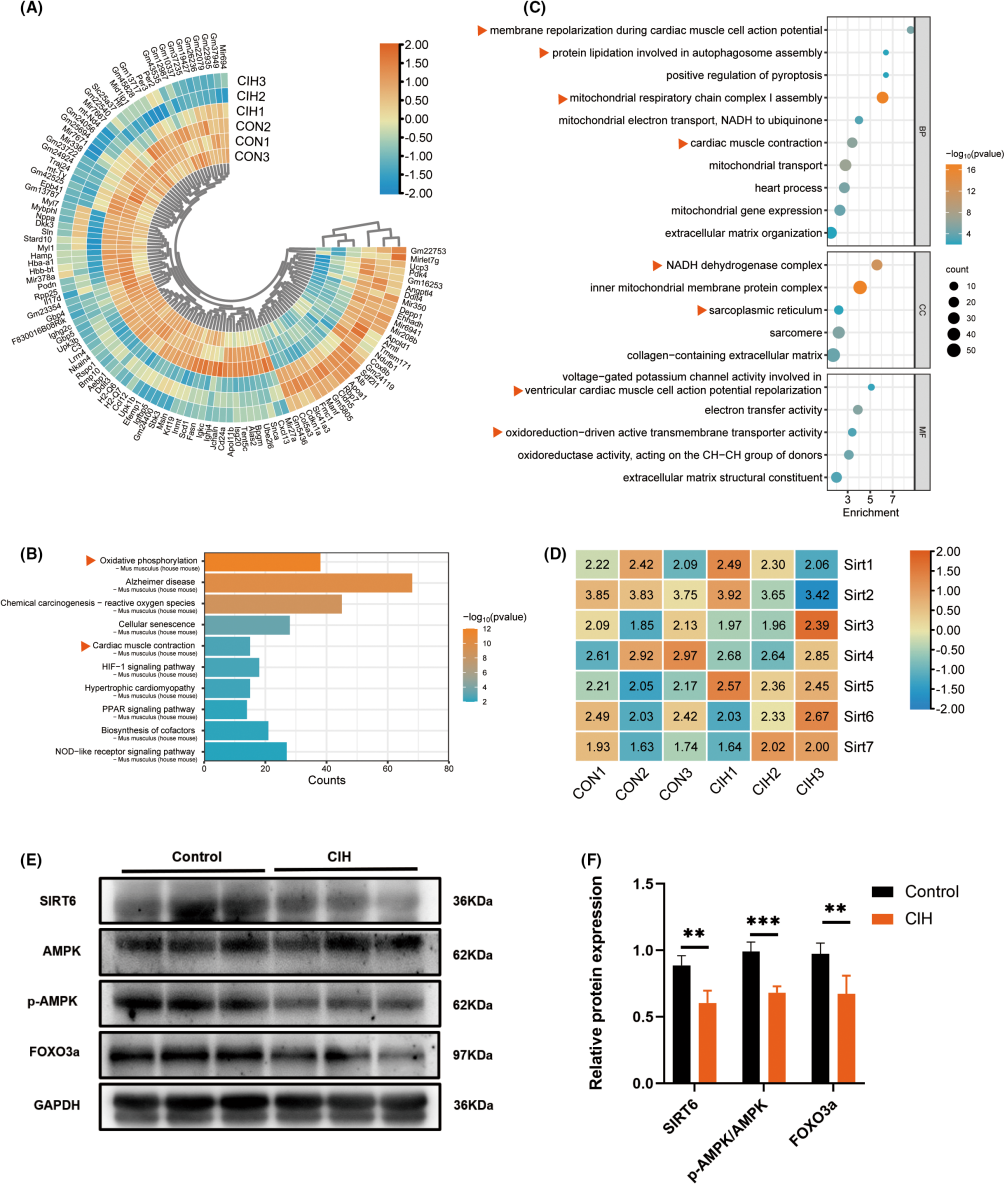
Mitochondrial dysfunction and impaired SIRT6 signalling might play critical roles in the pathogenesis of CIH‐induced cardiac injury. (A) Heatmap representing the selected DEGs (CIH vs. CON). A total of 114 top genes were ranked by Log_2_(FC). (B) Kyoto Encyclopedia of Genes and Genomes (KEGG) enrichment analysis of DEGs. (C) Dot‐plot representing the Gene Ontology (GO) analysis of DEGs. The longitudinal axis represents enriched GO function classifications, which were divided into three major categories: biological process (BP), molecular function (MF) and cellular component (CC). (D) Heatmap representing the gene of sirtuin (SIRT) family members (CIH vs. CON). (E, F) Western blotting and quantitative analysis of myocardial SIRT6, p‐AMPK/AMPK and FOXO3a in mice (*n* = 5/group). Data were presented as Mean ± SD. **p < 0.01 and ***p < 0.001 versus Normoxia.

The data set of GSE138008 is an atrial mRNA profile of C57BL/6J mice, which includes 3 samples of CIH model and 3 samples from the control group (CON). Mice were housed in customized cages to deliver CIH, during which hypoxic events occurred at a rate of one event per 180 s. Mice in the control group were exposed to normoxic air at all times.^20^ To investigate the mechanisms of CIH‐induced cardiac injury, a total of 18,456 genes were investigated in the bioinformatics analysis of this dataset and 1967 DEGs were identified for GO and KEGG analysis. The top 114 genes, including 30 up‐regulated DEGs and 84 down‐regulated DEGs were ranked by Log_2_(FC) (Figure 1A). An integrated bioinformatics platform (www.bioinformatics.com.cn) was employed to visualize the GO and KEGG enrichment. We found that the DEGs were mainly enriched in NADH dehydrogenase complex, inner mitochondrial membrane protein complex and sarcoplasmic reticulum (cellular component, CC). Further analysis showed that membrane repolarization during cardiac muscle cell action potential, protein lipidation involved in autophagosome assembly, mitochondrial respiratory chain complex I assembly, cardiac muscle contraction (biological process, BP) and voltage‐gated potassium channel activity involved in ventricular cardiac muscle cell action potential repolarization and oxidoreduction‐driven active transmembrane transporter activity (molecular function, MF) were significantly enriched (Figure 1C). KEGG analysis showed significant enrichment of the oxidative phosphorylation and cardiac muscle contraction (organismal systems) (Figure 1B). These results suggested that mitochondrial dysfunction‐induced oxidation–reduction imbalance might be a vital contributor to CIH‐induced cardiac injury.

Additionally, heatmap of genes involved in the sirtuin family were plotted with TBtools software (version 1.129) (Figure 1D). Although the gene expression differences among all members of the sirtuin family were not significant, we found a marked decrease in myocardial SIRT6 expression in CIH group (Figure 1E,F). Compared to the control group, the levels of AMPK phosphorylation and FOXO3a were also decreased in CIH group (Figure 1E,F), indicating that reduced SIRT6‐AMPK‐FOXO3a signalling might play a role in CIH‐associated cardiac injury.

### 3.2 BBR dose‐dependently inhibited CIH‐induced myocardial structural remodelling and cardiac dysfunction

**FIGURE 2:**
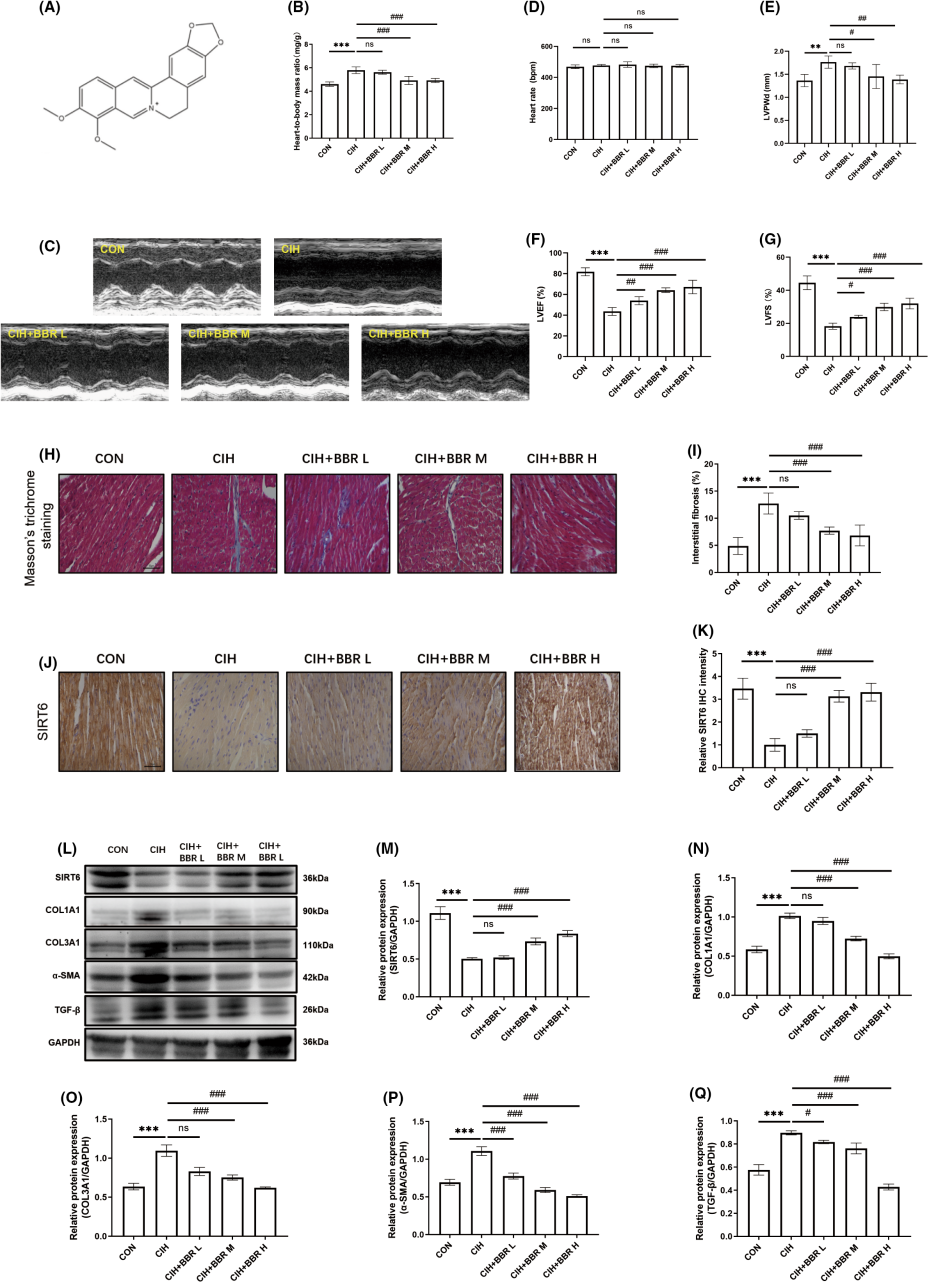
BBR dose‐dependently inhibited CIH‐induced myocardial structural remodelling and cardiac dysfunction. (A) Chemical structure of BBR (C_20_H_18_NO_4_^+^, 336.4 g/mol). (B) Heart to body mass ratio (*n* = 5/group). (C) Representative M‐mode parasternal long‐axis echocardiographic images (*n* = 5/group). (D) Heart rate during echocardiographic measurement (*n* = 5/group). (E–G) Left ventricular end‐diastolic posterior wall thickness (LVPWd), left ventricular ejection fraction (LVEF) and left ventricular fractional shortening (LVFS) (*n* = 5/group). (H, I) Representative Masson's trichrome staining images (scale bar = 50 μm) and quantitative analysis of interstitial fibrosis (*n* = 5/group). (J, K) Representative immunohistochemical staining images of SIRT6 (scale bar = 50 μm) and quantitative analysis of SIRT6 (*n* = 5/group). (L–Q) Representative western blot image and relative expression of myocardial SIRT6, COL1A1, COL3A1, α‐SMA and TGF‐β (*n* = 4/group). Data were presented as Mean ± SD. **p < 0.01 and ***p < 0.001 versus CON; ^#^ *p* < 0.05, ^##^ *p* < 0.01 and ^###^ *p* < 0.001 versus CIH. BBR L, BBR M and BBR H indicated that the mice were treated with BBR at the doses of 50, 100 and 200 mg/kg, respectively. ns, not significant.

To examine the effects of BBR (Figure 2A) treatment on CIH‐induced cardiac dysfunction and myocardial fibrosis, three gradient concentrations of BBR (50, 100, and 200 mg/kg) groups were established. We found no significant difference in heart rate among all groups (Table 1). Compared with the CON group, blood pressure and heart‐to‐body mass ratio increased significantly in the CIH group (Table 1 and Figure 2B). BBR treatment ameliorated these alterations in a concentration‐dependent manner (Table 1 and Figure 2B). In addition, echocardiographic results showed that BBR significantly attenuated cardiac dysfunction, as evidenced by the concentration‐dependent improvement in left ventricular ejection fraction (LVEF), left ventricular fractional shortening (LVFS) and left ventricular diastolic posterior wall thickness (LVPWd) in BBR‐treated group (Figure 2C–G). We found no significant difference in heart rate during echocardiographic measurement among all groups (Figure 2D). Moreover, our results revealed that berberine dose‐dependently reduced myocardial fibrosis, which was supported by a decrease in fibrosis area (Figure 2H,I) and reduced expression of fibrosis‐related markers (COL1A1, COL3A1, TGF‐β and α‐SMA, Figure 2L,N–Q). Additionally, the results of immunohistochemistry and western blot analyses demonstrated the inhibition of SIRT6 expression in the CIH group, while BBR treatment markedly activated SIRT6 signalling in a concentration‐dependent manner (Figure 2J–M).

**TABLE 1:** Physiological characteristics.

Variables	CON	CIH	CIH+BBR L	CIH+BBR M	CIH+BBR H
Heart rate, bpm	489 ± 12.4	504 ± 12.1	506 ± 14.3	492 ± 13.2	495 ± 16.6
Systolic blood pressure, mmHg	122 ± 11.9	160 ± 7.86*	153 ± 5.76^**ns**^	148 ± 10.37^#^	146 ± 10.07^#^
Diastolic blood pressure, mmHg	76 ± 5.03	88 ± 11.94*	87 ± 6.5^**ns**^	77 ± 7.2^#^	78 ± 8.07^#^

Abbreviation: ns, not significant.

* *p* < 0.05 versus the control group.

^#^ *p* < 0.05 versus the CIH group.

### 3.3 SIRT6 overexpression attenuated CIH‐induced myocardial dysfunction and myocardial structural remodelling

**FIGURE 3:**
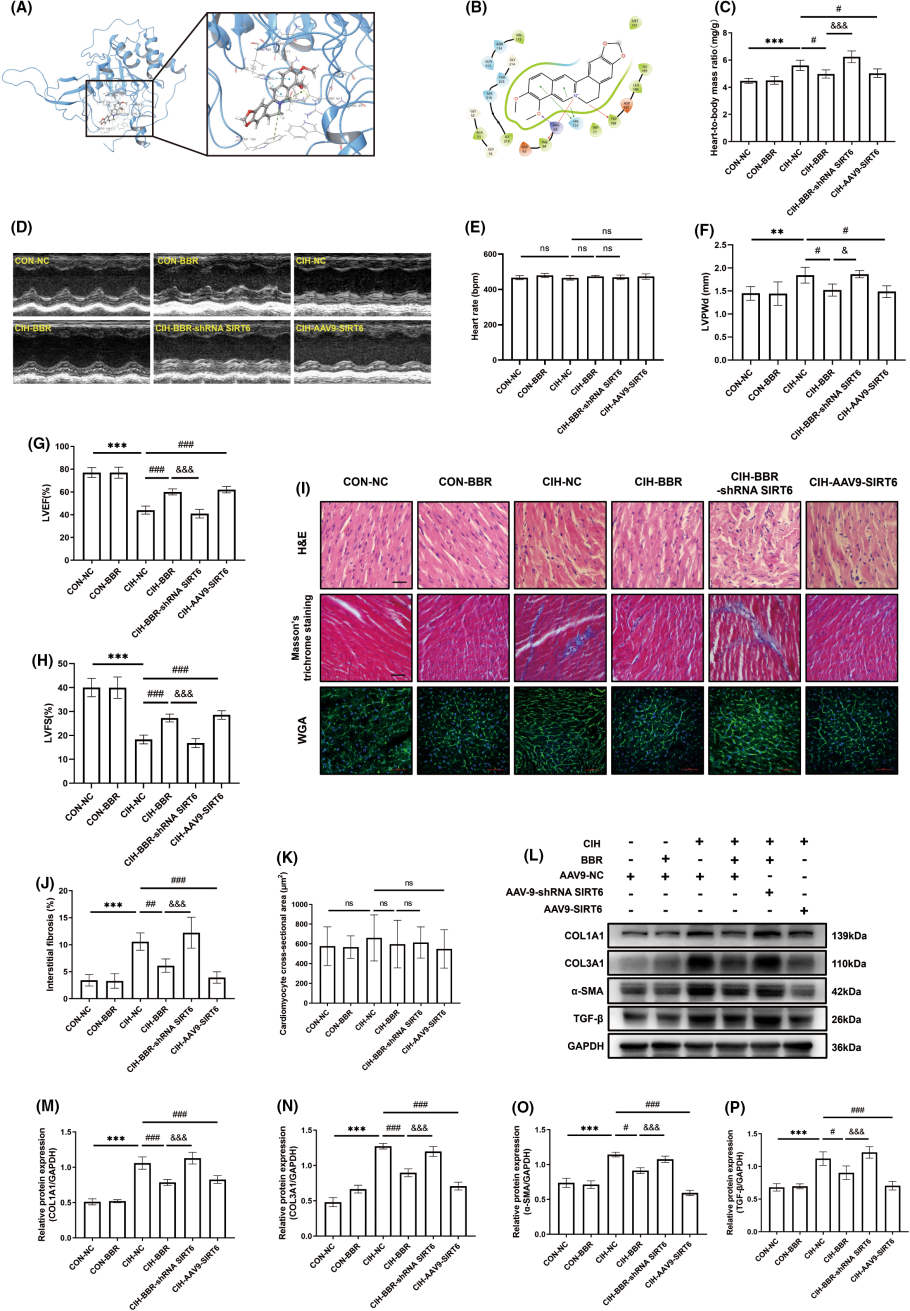
SIRT6 overexpression attenuated CIH‐induced myocardial dysfunction and myocardial structural remodelling. (A) Molecular docking of ligand BBR into SIRT6 target site (PDB ID: 6QCD) by using Schrodinger suite 2019–1. (B) 2D‐interaction pose of inhibitors illustrating hydrogen bonds (purple), pi‐cation (red) and pi‐pi stacking (green) formed with amino acid residues at the binding pocket of SIRT6. (C) Heart to body mass ratio (*n* = 5/group). (D) M‐mode of representative parasternal long‐axis echocardiographic images (*n* = 5/group). (E) Heart rate during echocardiographic measurement (*n* = 5/group). (F–H) Left ventricular end‐diastolic posterior wall thickness (LVPWd), left ventricular ejection fraction (LVEF) and left ventricular fractional shortening (LVFS, *n* = 5/group). (I) Representative H&E staining images (top, scale bar = 50 μm), Masson's trichrome staining images (middle, scale bar = 50 μm) and WGA staining images (bottom, scale bar = 50 μm, *n* = 5/group). (J) Quantitative analysis of interstitial fibrosis (*n* = 5/group). (K) Quantitative analysis of cardiomyocyte cross‐sectional area (*n* = 5/group). (L–P) Representative western blot image and relative expression of myocardial COL1A1, COL3A1, α‐SMA and TGF‐β (*n* = 4/group). Data were presented as Mean ± SD. **p < 0.01 and ***p < 0.001 versus CON+CN; ^#^ *p* < 0.05, ^##^ *p* < 0.01 and ^###^ *p* < 0.001 versus CIH + NC; ^&^ *p* < 0.05 and ^&&&^ *p* < 0.001 versus CIH + BBR; ns, not significant.

Based on the molecular docking analysis (Figure 3A,B), we found that BBR binds to the active site of SIRT6 by forming a hydrogen bond with an arginine residue (ARG‐65), pi‐cation with two amino acid residues (PHE‐64 and TRP‐188) and pi‐pi stacking interactions with a nearby amino acid residue (HIS‐133). To further investigate the role of SIRT6 in the beneficial effects of BBR, we utilized adeno‐associated virus to create cardiac‐specific SIRT6‐knockdown or overexpressed mice. In view of the data acquired in Figure 2, a concentration of 100 mg/kg BBR was selected in the subsequent experiments. As shown in Table 2, no significant difference in heart rate was found among these groups. Compared with the CON‐NC group, blood pressure and heart‐to‐body mass ratio were comparative in CON‐BBR group (Table 2 and Figure 3C). SIRT6 overexpression significantly attenuated CIH‐induced elevated blood pressure and increased heart‐to‐body mass ratio (Table 2 and Figure 3C). In addition, SIRT6 overexpression reduced left ventricular dysfunction (compared with the CIH group, Figure 3D,F–H). Meanwhile, overexpression of SIRT6 reduced myocardial fibrosis as evidenced by a decrease in fibrosis area (Figure 3I,J) as well as fibrosis‐related markers (Figure 3L–P). Nevertheless, SIRT6 knockdown inhibited the protective effects of BBR. The cross‐sectional area of cardiomyocytes was comparative among all these groups (Figure 3I,K). No significant changes were found in heart rate during the measurement of cardiac performance among these groups (Figure 3E). These findings suggested that SIRT6 overexpression ameliorated myocardial dysfunction and structural remodelling triggered by CIH in mice. Additionally, BBR exerted these beneficial effects in a SIRT6‐dependent manner.

**TABLE 2:** Physiological characteristics.

Variables	CON‐NC	CON‐BBR	CIH‐NC	CIH‐BBR	CIH‐BBR‐shRNA SIRT6	CIH‐AAV9‐SIRT6
Heart rate, bpm	477 ± 15.4	480 ± 10.6	495 ± 14.7	482 ± 15.3	496 ± 14.6	485 ± 13.9
Systolic blood pressure, mmHg	114 ± 5.49	117 ± 8.22	177 ± 7.24*	135 ± 6.68^#^	179 ± 5.93^&^	141 ± 4.97^#^
Diastolic blood pressure, mmHg	63 ± 2.88	64 ± 4.63	75 ± 5.47*	64 ± 4.96^#^	77 ± 2.93^&^	69 ± 2.48^#^

* *p* < 0.05 versus the control group.

^#^ *p* < 0.05 versus the CIH group.

^&^ *p* < 0.05 versus the CIH + BBR group.

### 3.4 BBR treatment enhanced the myocardial antioxidant capacity in CIH‐induced mice via SIRT6 signalling

**FIGURE 4:**
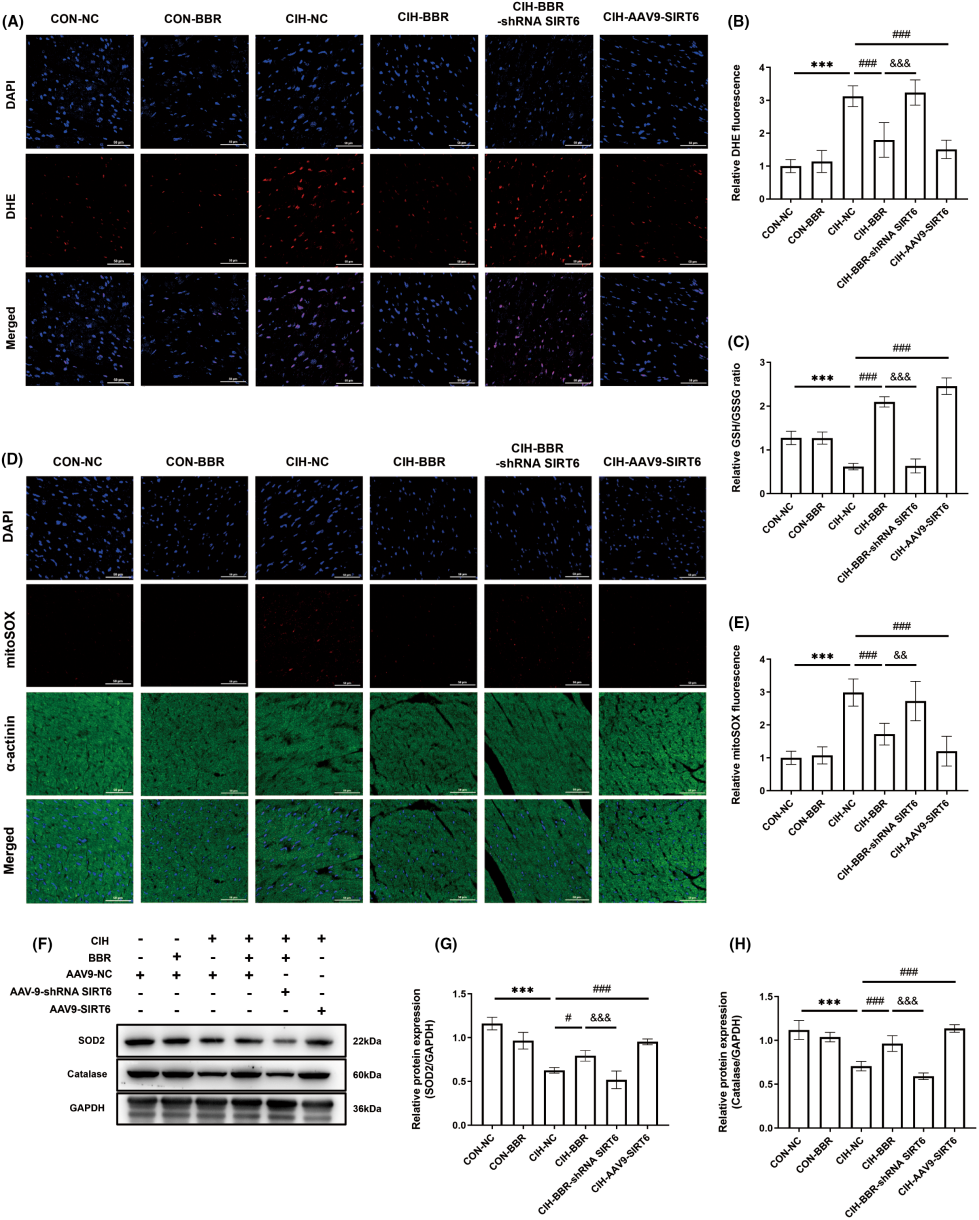
BBR treatment enhanced the myocardial antioxidant capacity in CIH‐induced mice via SIRT6 signalling. (A, B) Representative images of reactive oxygen species (ROS) (red) by dihydroethidium staining (bar = 50 μm) and quantitative analysis of mean intensity (*n* = 5/group). (C) Relative GSH/GSSG ratio (*n* = 5/group). (D, E) Representative fluorescence images of MitoSOX (red) in myocardial tissue (bar = 50 μm) and relative MitoSOX fluorescence intensity (*n* = 5/group). (F–H) Representative western blot image and relative expression of myocardial SOD2 and Catalase (*n* = 4/group). Data were presented as Mean ± SD. ***p < 0.001 versus CON+CN; ^#^ *p* < 0.05 and ^###^ *p* < 0.001 versus CIH + NC; ^&&^ *p* < 0.01 and ^&&&^ *p* < 0.001 versus CIH + BBR.

In comparison to the control group, CIH notably increased myocardial oxidative stress levels, as evidenced by increased fluorescence intensity of DHE and MitoSOX (Figure 4A,B,D,E), as well as decreased GSH/GSSG ratio (Figure 4C). BBR and SIRT6 overexpression obviously reversed these adverse effects of CIH, and these beneficial effects of BBR were abolished by SIRT6 knockdown (Figure 4A–E). Next, we detected the changes in the protein levels of antioxidant enzymes. Western blot analysis showed that the expression levels of SOD1, SOD2 and Catalase were significantly down‐regulated in CIH group (Figure 4F–H), which were reversed by BBR treatment or SIRT6 overexpression. In contrast, the beneficial effects of BBR were inhibited by SIRT6 knockdown (Figure 4F–H). These data suggested that BBR protected against oxidative stress in a SIRT6‐dependent manner.

### 3.5 BBR treatment activated myocardial AMPK‐FOXO3a pathway via SIRT6 signalling

**FIGURE 5:**
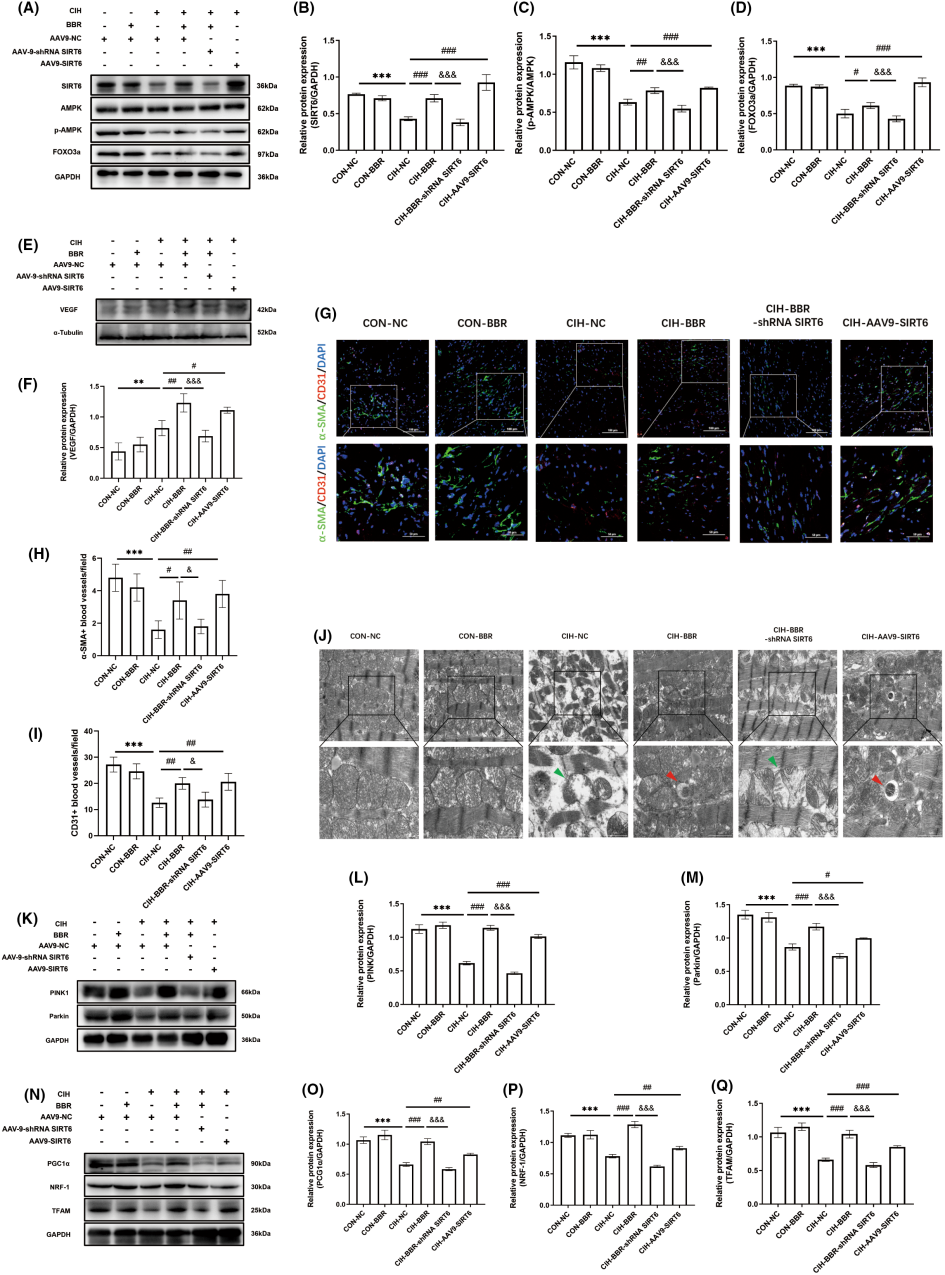
BBR treatment promoted microvascular formation, improved mitochondrial autophagy and biogenesis via SIRT6 signalling. (A–D) Representative western blot image and the associated quantitative analysis of myocardial SIRT6 expression, the AMPK phosphorylation level and FOXO3a expression (*n* = 4/group). (E, F) Representative western blot image and relative expression of myocardial VEGF (*n* = 4/group). (G–I) Representative fluorescence images of α‐SMA (green)‐ and CD31 (red)‐positive vessels in myocardial tissue (upper bar = 100 μm, lower bar = 50 μm) and the quantitative analysis (*n* = 5/group). (J) TEM images of myocardial tissue. Green arrowheads: damaged mitochondria. Red arrowheads: autophagosomes carrying mitochondria (upper scale = 2 μm, lower scale = 1 μm, *n* = 5/group). (K–M) Representative western blot image and relative expression of myocardial PINK1 and Parkin (*n* = 4/group). (N–Q) Representative western blot image and relative myocardial protein expressions of PGC1α, NRF‐1 and TFAM (*n* = 4/group). Data were presented as Mean ± SD. **p <0.01 and ***p < 0.001 versus CON + CN; ^#^ *p* < 0.05, ^##^ *p* < 0.01 and ^###^ *p* < 0.001 versus CIH + NC; ^&^ *p* < 0.05 and ^&&&^ *p* < 0.001 versus CIH + BBR.

To investigate the underlying mechanisms, we determined the potential downstream targets of SIRT6 by employing AAV9 vector carrying SIRT6 or SIRT6 shRNA (directed by the cardiac‐specific c‐TNT promoter) as described in our previous study.^12^, ^25^, ^26^ As shown in Figure 5A,B, compared with the CIH + AAV9‐NC group, the relative expression of SIRT6 in the CIH + AAV9‐SIRT6 group was significantly increased (0.93 ± 0.11 vs. 0.43 ± 0.03). Meanwhile, SIRT6 protein level exhibited a markedly decrease in CIH‐BBR‐shRNA SIRT6 group (0.38 ± 0.05 vs. 0.71 ± 0.05, compared with the CIH + BBR + AAV9‐NC group), suggesting that, in the present study, the AAV9 transduction system achieved satisfactory effects. As shown in Figure 5A–D, CIH decreased the protein expression of SIRT6, down‐regulated the AMPK‐FOXO3a signalling (compared with the CON‐NC group). Meanwhile, BBR treatment or SIRT6 overexpression not only enhanced the expression of SIRT6, but also re‐activated AMPK phosphorylation as well as FOXO3a level (compared with the CIH group, Figure 5A–D). Additionally, SIRT6 knockdown inhibited BBR‐mediated activation of SIRT6‐AMPK‐FOXO3a signalling (Figure 5A–D). These data revealed that BBR activated the AMPK‐FOXO3a signalling pathway by upregulating SIRT6.

### 3.6 BBR promoted myocardial microvascular formation via SIRT6 signalling

Previous study has indicated that hypoxia promotes the formation of small arteries and capillaries. Here, we evaluated the arterio‐genesis and angiogenesis by analysing smooth muscle cell marker α‐SMA and endothelial cell marker CD31 immunofluorescence. The level of vascular endothelial growth factor (VEGF) was also quantified. Compared with the CON‐NC group, CIH increased the protein expression of VEGF (Figure 5E,F). Interestingly, BBR or SIRT6 overexpression both significantly increased the expression of VEGF compared with the CIH‐NC group, while this effect was abolished by SIRT6 knockdown (Figure 5E,F). In comparison to the CON‐NC group, the numbers of both α‐SMA‐ and CD31‐positive vessels were decreased in the CIH group (Figure 5G–I). BBR and SIRT6 overexpression notably reversed the adverse effect of CIH, and the ameliorative effect of BBR was abolished by SIRT6 knockdown (Figure 5G–I). These data indicated that BBR improved microvascular formation possibly though SIRT6 signalling.

### 3.7 BBR improved mitochondrial autophagy and biogenesis via SIRT6 signalling

Considering the critical role of mitochondria in sustaining the energy requirements of cardiomyocytes,^31^ we further evaluated myocardial mitochondrial function with a focus on mitochondrial morphology, mitophagy and mitochondrial biogenesis. Electron microscopic examination showed that the control group displayed well‐developed mitochondria with integral membrane and cristae (Figure 5J). However, the CIH group displayed obvious degenerative changes in mitochondrial morphology, including abnormal shape, swelling and disorganized cristae (Figure 5I). As shown in Figure 5K–M, CIH significantly down‐regulated PINK1 and Parkin expression (compared with the CON‐NC group). BBR administration or SIRT6 overexpression improved mitophagy signalling of PINK1‐Parkin (compared with the CIH group). Transmission electron microscopy images also showed that groups of BBR treatment or SIRT6 overexpression exhibited obvious autophagosomes carrying mitochondria and alleviated mitochondrial morphological abnormality (Figure 5J). Furthermore, compared with the CIH‐BBR group, the PINK1‐Parkin signalling was significantly inhibited in the CIH‐BBR‐shRNA SIRT6 group (Figure 5K–M). Next, the mitochondrial biogenesis‐related proteins PGC1α, NRF‐1 and TFAM were detected. Compared with the CON‐NC group, CIH group down‐regulated the protein expressions of PGC1α, NRF‐1 and TFAM, which was reversed by BBR or SIRT6 overexpression (Figure 5N–Q). However, these effects exerted by BBR were abolished by SIRT6 knockdown (Figure 5N–Q). These results indicated that BBR effectively improved myocardial mitophagy and mitochondrial biogenesis through SIRT6 signalling.

## 4 DISCUSSION

**FIGURE 6:**
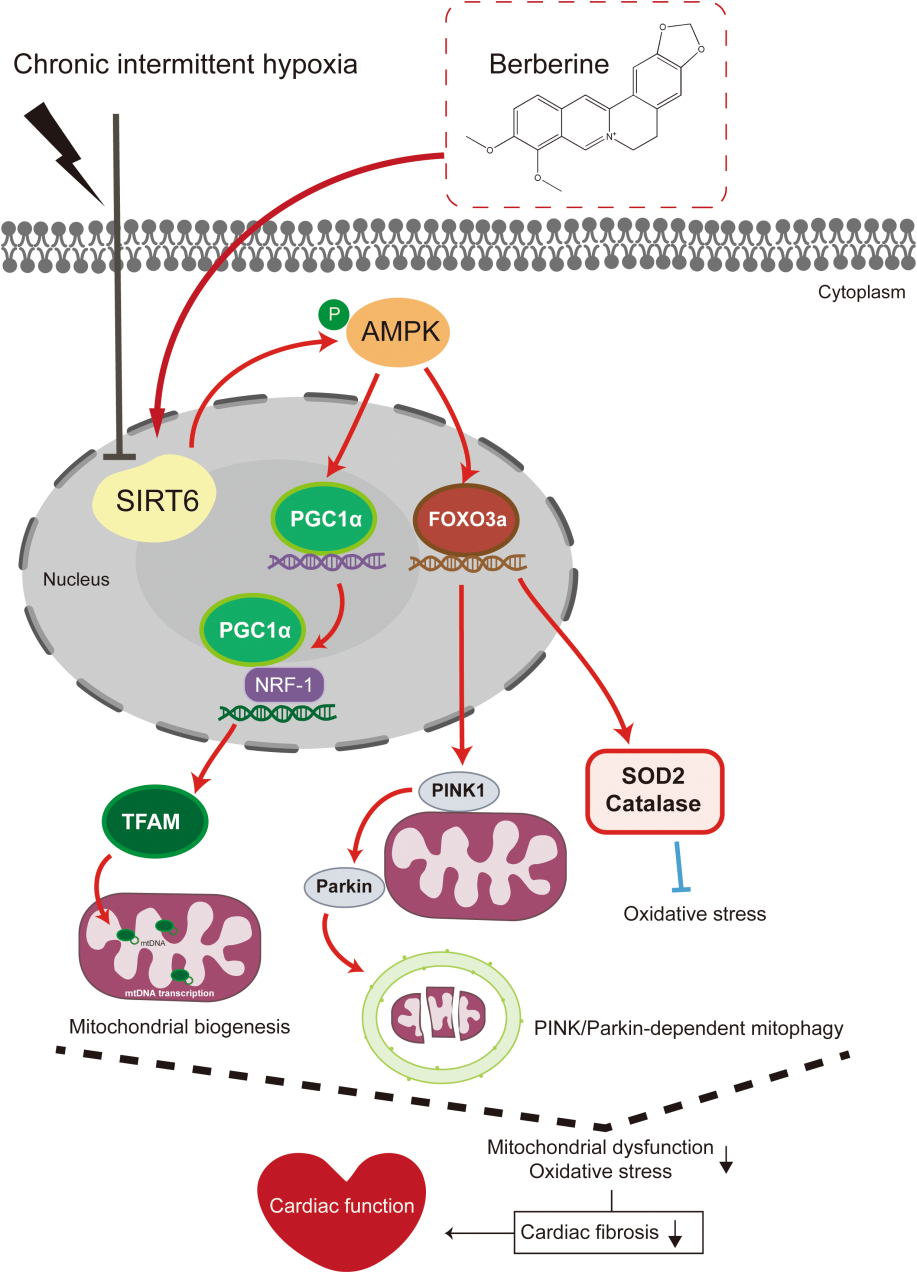
Schematic illustration of this study. Crucial roles of SIRT6 in regulation of oxidative stress, mitochondrial biogenesis and mitophagy and the protective effects of BBR. SIRT6 plays a protective role against CIH‐induced myocardial injury. BBR protects against CIH‐induced oxidative stress and cardiac fibrosis by improving mitochondrial biogenesis and PINK1‐Parkin‐dependent mitophagy via the SIRT6‐AMPK‐FOXO3a signalling pathway.

Several major findings have been achieved in this study. First, downregulation of SIRT6 signalling was found in CIH‐injured myocardium. Second, cardiac SIRT6 signalling was shown to play an important role in protecting myocardium against CIH‐induced myocardial oxidative stress and fibrosis. Third, BBR attenuated CIH‐induced cardiac damage by promoting mitochondrial autophagy and biogenesis via the SIRT6‐AMPK‐FOXO3a signalling pathway (Figure 6).

Studies have demonstrated that CIH triggers secondary sympathetic activation, oxidative stress, and systemic inflammation, resulting in adverse effects on the heart including contractile dysfunction, atrial fibrillation, and even heart failure.^32^, ^33^, ^34^ To date, there is still a lack of efficient therapeutic approaches. Thus, it is necessary to further elucidate the molecular mechanism of CIH in inducing myocardial injury and to develop new therapeutic targets. Members of the VEGF family and their receptors play a crucial role as regulators of angiogenesis and vessel maintenance.^35^ It has been revealed that CIH up‐regulates the expression of VEGF via the activation of the oxygen‐dependent transcription factor HIF‐1α, which in turn induces vascular smooth muscle cell proliferation and vascular remodelling.^36^ A prior study by Visniauskas et al. showed that myocardial angiogenesis and capillary density were both decreased from the third to the fifth weeks of CIH exposure, accompanied by delayed increase in both gene and protein expression of VEGF.^37^ They further demonstrated that this discrepancy was related to the classic interaction between VEGF and kallikrein‐kinin system (KKS) components, and their temporal expressions were not sufficient to stimulate the formation of new blood vessels. This could partially explain our results. In the present study, we showed that CIH obviously decreased the arteriolar and capillary density, while the expression of VEGF was significantly increased in CIH group. Previous study has also showed that VEGF expression was notably increased in hypoxia mice while the numbers of α‐SMA+ and CD31+/vWF+ positive cells were significantly decreased, which was consistent with our results.^38^ The interplay of myocardial arterio‐genesis and angiogenesis as well as angiogenic factors during CIH exposure warrants further investigation.

Among the sirtuin family members, SIRT1, SIRT3 and SIRT6 have attracted much attention as the potential cardioprotective modulators against inflammation, vascular remodelling, cardiomyopathy and the development of atherosclerotic plaques.^39^ Previously, we have reported the critical role of SIRT6‐AMPK axis in reducing mitochondrial division, enhancing mitochondrial biogenesis and mitochondrial autophagy in diabetic cardiomyopathy (DCM).^12^ In addition, Wang et al. found that SIRT6 reduced the level of cellular oxidative stress to protect myocardial infarction in ischemic heart disease by upregulating AMPK‐FOXO3a axis, and then activating the expression of downstream antioxidant coding genes.^10^ Numerous studies have shown that AMPK‐FOXO3a signalling is involved in the upregulation of antioxidant enzymes in response to oxidative stress.^40^, ^41^ Here, we found that SIRT6‐AMPK‐FOXO3a signalling pathway were down‐regulated in CIH‐induced mouse ventricular tissue (Figure 1), while in the bioinformatics analysis, there were no significant differences in mRNA levels among seven members of the sirtuin family (SIRT1‐SIRT7). We speculate that the inconsistency may be due to the regulatory mechanisms regarding the translational or post‐translational modifications of these key molecules. Besides, we noticed a prior RNA‐seq study by Castro‐Grattoni et al. showed that CIH‐induced upregulation of stress‐response genes were involved in cardioprotection (including SIRT6) in young female mice instead of aged ones.^42^ It is noted that there are real differences between males and females in the presentation, pathophysiology and comorbidities regarding to obstructive sleep apnoea (OSA). Some published studies suggest a higher risk of cardiovascular comorbidity^43^ and type 2 diabetes^44^ in males with OSA. Lin et al. revealed that atrial fibrillation burden is a sex‐specific risk factor for OSA and is limited to male.^45^ Moreover, women with both AF and OSA have a lower AF burden than men, despite being older and having similar OSA severity and body habitus.^45^ Thus, we think this could also result in the difference of these studies. Besides, the experimental procedures and duration of CIH are also different between our study and the work by Castro‐Grattoni.^42^ Nonetheless, we found that SIRT6 overexpression attenuated CIH‐induced myocardial dysfunction, myocardial structural remodelling and enhanced the myocardial antioxidant capacity. These data demonstrated that impaired SIRT6‐AMPK signalling contributed greatly to CIH‐induced cardiac dysfunction and remodelling. The influence of age and sex on the cardiac response to CIH exposure deserves further investigation.

BBR is recognized for its multiple cardiovascular protective effects and can be used to treat diabetes, hypertension, arrhythmias and other conditions.^14^, ^16^ An increasing number of studies suggest that BBR possesses profound antioxidative property.^15^ Our group and others have showed that BBR can positively regulate the synthesis of cellular antioxidant enzymes (SOD2 and Catalase), and acts as a cardiovascular protector,^19^, ^40^, ^46^ although the underlying mechanisms have not been fully elucidated. As a critical energy sensor, AMPK is essential to maintain the body's energy balance. It has been reported that SIRT1, SIRT3 and SIRT6 play crucial roles in regulating mitochondrial biogenesis and oxidative stress by modulating AMPK.^5^ Moreover, these three members of the sirtuin family have also been demonstrated to regulate oxidative stress through FOXO3a.^10^, ^41^, ^47^, ^48^, ^49^ Previous work by our group has reported that BBR improves myocardial ischemia reperfusion injury by reducing oxidative stress and inflammation response in a SIRT1‐dependent manner.^19^ Other researches have also shown that BBR can increase the protein levels of SIRT1 and SIRT3 to regulate autophagy and mitochondrial biogenesis in cardiomyocytes, which maintains a balance in mitochondrial quality control and reduces DOX‐induced cardiotoxicity.^18^ Nevertheless, the potential cardioprotective effects of BBR on CIH and the role of SIRT6 signalling remain uncovered. Here, we showed that BBR treatment ameliorated myocardial damage and structural remodelling caused by CIH (Figure 2). Furthermore, SIRT6 knockdown restricted the AMPK‐FOXO3a signalling activity and the benefits of BBR administration (Figures 3, 4, 5). These findings were in line with the previous reports showing the regulatory effect of SIRT6 on AMPK signalling,^12^, ^50^ suggesting that AMPK‐FOXO3a signalling at least in part, mediated the beneficial effects of BBR‐induced SIRT6 activation.

Previous studies have shown that BBR exerts cardiovascular protective effect by improving mitochondrial function.^51^, ^52^, ^53^ Our recent study demonstrated that impaired SIRT6‐AMPK signalling played a key role in cardiac dysfunction by damaging mitochondrial dynamics and mitophagy.^25^ Additionally, several studies have highlighted the role of FOXO3a in maintaining mitochondrial homeostasis by regulating PINK‐Parkin‐mediated mitophagy.^54^ It is worth noting that CIH‐induced myocardial oxidative stress damage is associated with the malfunction of mitophagy and mitochondrial biogenesis. Clinical data indicated that patients with OSA exhibited elevated levels of oxidative stress markers and reduced mitochondrial DNA copy numbers, which correlated with the severity of the condition.^55^, ^56^ Meanwhile, it has been found that myocardial cells injured by CIH are susceptible to oxidative stress and mitochondrial dysfunction, which results in the accumulation of excessive iron, while activation of mitochondrial autophagy could explicitly lower iron accumulation in the mitochondria, thereby attenuating CIH‐induced cardiac dysfunction and structural remodelling.^57^ On the other hand, enhancing early adaptive changes in mitochondrial biogenesis through activation of the PGC1α/Akt signalling pathway exerts a protective effect against CIH‐induced cardiac injury.^58^ In the current study, we found that the level of AMPK phosphorylation and the expression of FOXO3a, mitophagy‐related molecules (PINK1 and Parkin), as well as mitochondrial biogenesis‐related markers (PGC1α, NRF‐1 and TFAM) were up‐regulated by SIRT6 overexpression and BBR treatment (Figure 5). Conversely, SIRT6 knockdown abrogated the beneficial effects of BBR on mitochondrial function. These data revealed that BBR promoted mitochondrial quality control through a SIRT6‐dependent pathway, and AMPK‐ FOXO3a axis probably participated in this process. Additionally, previous studies showed that BBR promotes the expression of VEGF by inhibiting the macrophage Wnt5a/β‐catenin pathway, and thus inducing angiogenesis in the infarcted heart.^59^ Another recent study suggested that overexpression of SIRT6 down‐regulated the acetylation level of H3K9ac, promoted myocardial angiogenesis, ameliorated mitochondrial damage and then improved myocardial remodelling.^60^ However, it is unclear whether BBR promotes angiogenesis and improves cardiac remodelling in CIH‐induced myocardium through SIRT6 signalling. In the present study, we showed that BBR treatment promoted myocardial angiogenesis, while the effects were abolished by SIRT6 knockdown (Figure 5). These results were partially coincided with the previous research showing that the long‐term dietary intake of pasta containing 3% barley β‐D‐glucan (P‐BBG) may simultaneously promote the angiogenesis and higher tolerance to oxidative stress due to enhanced endothelial expression of VEGF/MnSOD signalling and Parkin.^61^ Meanwhile, it has been observed that enhancing the overall cellular antioxidant capacity may also result in the upregulation of VEGF and Parkin within the cardiovascular system, as indicated by the positive regulatory influence of MnSOD on VEGF or Parkin signalling.^61^, ^62^ The intricate interaction among these molecules deserves further investigation.

We acknowledge that there are several limitations in this study. Despite our results that SIRT6 plays a protective effect in the therapeutic actions of BBR, further research is required to investigate whether the effects of BBR in CIH‐induced cardiac injury are related to other members of the sirtuin family (especially SIRT1 and SIRT3). On the other hand, while the AAV9 virus effectively regulated cardiac SIRT6 expressions, we did not assess the transduction efficiency of the virus. Meanwhile, the potential for non‐specific effects of the virus on different cardiac cell types or even other organs cannot be discounted. Animal models featuring cardiac‐specific overexpression or knockout of SIRT6 are unquestionably necessary to substantiate the conclusion. Additionally, although the present study provides in vivo evidence that BBR might serve as a promising cardioprotective agent against CIH‐induced myocardial dysfunction, its direct effects on the pathophysiological mechanisms of OSA, including upper respiratory airway resistance, upper respiratory tract oedema and hypertrophied tonsils, are largely unknown. These aspects deserve further basic and clinical research.

Taken together, the present study reveals the therapeutic effects and mechanisms of BBR against CIH‐induced cardiac injury. We demonstrate that BBR ameliorates myocardial oxidative stress and fibrosis by promoting mitophagy and mitochondrial biogenesis. Importantly, the SIRT6‐AMPK‐FOXO3a signalling pathway plays a vital role in this process. Activation of SIRT6 signalling may be a novel approach to treat CIH‐induced cardiac dysfunction. Further translational studies are needed regarding the clinical applications of BBR in this circumstance.

## AUTHOR CONTRIBUTIONS

**Zijun Zhou:** Data curation (equal); formal analysis (equal); investigation (equal); methodology (equal); writing – original draft (equal); writing – review and editing (equal). **Qiusheng Zhao:** Data curation (equal); formal analysis (equal); investigation (equal); methodology (equal); resources (equal); visualization (equal); writing – original draft (equal). **Yuting Huang:** Data curation (equal); formal analysis (supporting); methodology (supporting); visualization (supporting); writing – original draft (supporting); writing – review and editing (supporting). **Shan Meng:** Investigation (supporting); methodology (supporting); visualization (supporting). **Xin Chen:** Formal analysis (supporting); investigation (supporting); methodology (supporting). **Guoxin Zhang:** Formal analysis (supporting); investigation (supporting); methodology (supporting); validation (equal); visualization (supporting). **Yanbang Chi:** Investigation (supporting); methodology (supporting); validation (supporting); visualization (supporting). **Dengyue Xu:** Investigation (supporting). **Zongtao Yin:** Funding acquisition (equal); supervision (equal); writing – review and editing (supporting). **Hui Jiang:** Supervision (supporting); writing – review and editing (supporting). **Liming Yu:** Conceptualization (equal); funding acquisition (supporting); supervision (equal); visualization (equal); writing – original draft (supporting); writing – review and editing (equal). **Huishan Wang:** Conceptualization (equal); funding acquisition (lead); project administration (lead); supervision (equal); writing – review and editing (supporting).

## CONFLICT OF INTEREST STATEMENT

The authors declare that they have no conflict of interests.

## CONTRIBUTION TO THE FIELD

Chronic intermittent hypoxia (CIH) is associated with an increased risk of multiple cardiovascular diseases. Although berberine (BBR) has been established as a potential cardioprotective agent, the effect and mechanism of BBR on CIH‐induced cardiomyopathy are still unknown. The present study was designed to determine the protective effects of BBR against CIH‐induced cardiac damage and to explore the molecular mechanisms with a focus on SIRT6 signalling. The data demonstrates that SIRT6 plays a protective role against the pathogenesis of CIH‐induced cardiac remodelling and oxidative stress. BBR protects against CIH‐induced myocardial injury by improving mitochondrial biogenesis and PINK1‐Parkin‐dependent mitophagy via the SIRT6‐AMPK‐FOXO3a signalling pathway. This study reveals new therapeutic effects of BBR and suggests that activation of SIRT6 signalling may be a novel approach to overcoming CIH‐induced cardiac dysfunction.

## Data Availability

The data that support the findings of this study are available from the corresponding author upon reasonable request.
